# The Aqueous Extract from the Stem Bark of *Garcinia lucida* Vesque (Clusiaceae) Exhibits Cardioprotective and Nephroprotective Effects in Adenine-Induced Chronic Kidney Disease in Rats

**DOI:** 10.1155/2021/5581041

**Published:** 2021-03-13

**Authors:** Christelle Stéphanie Sonfack, Elvine Pami Nguelefack-Mbuyo, Jacquy Joyce Kojom, Edwige Laure Lappa, Fernande Petingmve Peyembouo, Christian Kuété Fofié, Tsabang Nolé, Télesphore Benoît Nguelefack, Alain Bertrand Dongmo

**Affiliations:** ^1^Laboratory of Animal Physiology and Phytopharmacology, Faculty of Science, University of Dschang, P.O. Box 67, Dschang, Cameroon; ^2^Department of Animal Biology and Physiology, Faculty of Sciences, University of Douala, P.O. Box 24157, Douala, Cameroon; ^3^Higher Institute of Environment Sciences, P.O. Box 16317, Yaounde, Cameroon

## Abstract

Chronic kidney disease (CKD) is a serious health problem with high morbidity and mortality, mainly attributable to cardiovascular risk. *Garcinia lucida* is traditionally used in Cameroon for the management of cardiovascular diseases. The aim of this study was to evaluate the cardioprotective and nephroprotective effects of the aqueous extract from the stem bark of *G*. *lucida* (AEGL). The in vitro antioxidant effect of AEGL was assessed at concentrations ranging 1–300 *μ*g/mL against DPPH, lipid peroxidation, and AAPH-induced hemolysis. The reducing power and phenolic and flavonoids contents were also determined. CKD was induced by intraperitoneal bolus injection of adenine (50 mg/kg/day) for 4 consecutive weeks to male Wistar rats. AEGL (150 and 300 mg/kg/day) or captopril (20 mg/kg/day) was concomitantly administered with adenine per os. Bodyweight and blood pressure were monitored at baseline and weekly during the test. At the end of the experiment, plasma creatinine, urea, AST, and ALT were quantified. Proteinuria, creatinine excretion, and creatinine clearance were also assessed. The effect on GSH, CAT, and SOD activity was evaluated in cardiac and renal homogenates. Sections of the heart and kidney were stained with hematoxylin and eosin. AEGL exhibited a potent in vitro antioxidant activity and was shown to possess a large amount of phenolic compounds. Adenine alone increased blood pressure, cardiac and kidney mass, proteinuria, protein to creatinine ratio, plasma creatinine, AST, and urea levels (*p* < 0.05, 0.01, and 0.001). Besides, the bodyweight and creatinine clearance were significantly reduced (*p* < 0.05 and *p* < 0.01). All these alterations were blunted by the plant extract, except the bodyweight loss. In addition, AEGL improved GSH levels and CAT and SOD activities. AEGL attenuated adenine-induced glomerular necrosis, tubular dilatation, and cardiac inflammation. AEGL exhibits cardioprotective and nephroprotective effects that may be ascribed to its antihypertensive and antioxidant activities.

## 1. Introduction

Chronic kidney disease (CKD) is commonly defined based on the value of estimated glomerular filtration rate (eGFR), with a 40% decline to a GFR or less than 60 ml/min/1.73 m^2^ for more than 3 months [1]. A broader definition of CKD has been proposed in 2012 by the Kidney Disease: Improving Global Outcomes (KDIGO) that takes into consideration structural, functional, and pathological abnormalities of the kidney [2] and not only functional alterations. Clinical signs of CKD include increased urinary protein excretion, reduced urinary creatinine, and increased serum or plasma creatinine [1, 3]. Reports from the global burden of disease (GBD) [4] clearly show that CKD is a growing global health problem with increasing incidence and prevalence in both developed and developing countries. It is associated with high morbidity and mortality, mainly attributable to elevated cardiovascular risk [4, 5].

In 2017, the worldwide prevalence of CKD was estimated at 9.1% with 1.2 million deaths, a number that has been projected to rise by 2040 to 2.2 million in a best-case scenario and up to 4.0 million in a worst-case scenario if effective measures are not put in place [4]. CKD has been ranked by GBD as the 12^th^ leading cause of death out of 133 conditions [4]. In Cameroon, the prevalence of CKD varied 3–14.1% and 10.0–14.2% in rural and urban areas, respectively [6].

The burden of CKD is worsened by the increased prevalence of hypertension and diabetes which play a key role in the morbidity and mortality associated to CKD [6, 7]. In addition, CKD in turn is a serious independent risk factor for the development of cardiovascular disease including hypertension [7, 8]. In fact, CKD and end-stage renal disease carry a 5-fold to 10-fold higher risk for developing cardiovascular disease compared to age-matched controls [8]. Cardiovascular disorders are the main causes of morbidity and mortality in patients with chronic kidney disease [1, 9]. These patients show high cardiovascular morbidity and mortality as a result of endothelial dysfunction, hypertension, and left ventricular hypertrophy [8, 10]. Oxidative stress and inflammation are two well-known important contributors in the pathophysiology of CKD [8, 11, 12], and the combination of oxidative stress, chronic inflammation, and endothelial dysfunction is recognized as a triad perpetuating the bidirectional vicious cycle between CKD and systemic complications [7].

Currently, there is no cure for most forms of CKD, and strategies to manage CKD rely on high blood pressure control through blockade of the renin-angiotensin system [1]. However, the effectiveness of this treatment is limited because it only delays end-stage kidney disease development which requires renal replacement therapy (RRT) such as dialysis or kidney transplantation [1]. Yet, in many countries, accessibility to, or affordability of RRT is restricted and even scarce, causing the death of 2.3–7.1 million adults [4, 6]. Therefore, the search for effective drugs that can successfully treat CKD is of paramount importance.

*Garcinia lucida* Vesque (Clusiaceae), commonly known as “essok” by the Cameroonian populations, is a small evergreen dioecious tree, reaching 25–30 cm in diameter at breast height and 12–15 m in height [13]. The stem bark of *G*. *lucida* is traditionally used to prevent food poisoning, cure stomach and gynecological pains, as well as snake bites [13]. Based on the ancestral knowledge, the plant is used in the south region of Cameroon for the management of cardiovascular ailments. Investigation of the pharmacological potential of *G*. *lucida* showed that alkaloids isolated from the stem bark exhibited trypanocidal and antileishmanial activities [14]. Extracts from *G*. *lucida* have also demonstrated cytotoxic, antimicrobial, and in vitro antioxidant activities [15, 16]. In a previous study, we showed that *G*. *lucida* prevented L-NAME-induced hypertension (unpublished data), but nothing is known about the effect of this plant on CKD. The present study was therefore undertaken to evaluate the antioxidant and the nephroprotective effects of the aqueous extract from the stem bark of *G*. *lucida* against adenine-induced chronic kidney disease in rats.

## 2. Materials and Methods

### 2.1. Plant Collection and Extraction

Fresh barks of *Garcinia lucida* were collected in Mvengue, in the south region of Cameroon in March 2017. The plant specimen was authenticated at the National Herbarium of Cameroon in comparison with an existing voucher specimen number 57193/HNC. The fresh stem bark was shade-dried and ground into a fine powder. The aqueous extract was prepared by bringing to boil 200 g of powder in 1.5 L distilled water for an hour. The residue was again decocted in 1.2 L distilled water for 1 h. Both filtrates were mixed and evaporated in an AISET YLD-2000 temperature controlled oven at 40°C. The extraction procedure yielded 37 g of aqueous extract, corresponding to an extraction yield of 18.5%.

### 2.2. Estimation of Total Phenolic Content

The total phenolic content was determined using the Folin–Ciocalteu method [17]. A calibration curve for gallic acid was used to estimate the amount of total phenolic content.

### 2.3. Estimation of Total Flavonoids

A modified method of Zhishen et al. [18] was used to estimate the total flavonoids content of extract from the stem bark of *G*. *lucida*. Briefly, 0.1 mL of aqueous extract (500 *μ*g/mL) was mixed with 30 *μ*L of 5% sodium nitrate and 1.4 ml distilled water and incubated at room temperature for 5 min. Then, 30 *μ*l of 10% aluminum chloride was added followed by 200 *μ*l of 0.1 M potassium hydroxide solution 6 min later. Optical densities were read at 510 nm. The flavonoids content was expressed as mg of quercetin per gram of sample.

### 2.4. Animals

Male Wistar rats aged 3-4 months and weighing 200–260 g were bred in the animal house of the Laboratory of Biology and Physiology of Animal Organism of the Faculty of Sciences, University of Douala-Cameroon, in plastic cages. Rats were maintained at a room temperature of 24 ± 2°C under natural light/dark cycle (∼12 h/12 h). They had free access to standard laboratory chow and tape water at libitum. The study was approved by the institutional ethic committee for research in human health of the University of Douala N 2043CEI-UDo/06/2019/T, and all experiments and procedures were performed in accordance with the guidelines on the protection of animals used for scientific purposes as described by the law 2010/63/EU of the European Parliament and of the Council of 22 September 2010.

### 2.5. In Vitro Antioxidant Tests

#### 2.5.1. DPPH Radical Scavenging Test

The radical scavenging ability of the aqueous extract from *G*. *lucida* was evaluated on 2,2-diphenyl-1-picrylhydrazyl (DPPH) as described by Nono et al. [19]. Ascorbic acid was used as standard. Briefly, a 0.16 mM DPPH solution was freshly prepared in methanol and incubated in the presence or absence of plant's extract (1–300 *μ*g/mL). All substances used were dissolved in methanol. The optical densities were read before (OD_1_) and 30 min after (OD_2_) adding DPPH at 515 nm using a Helios Epsilon spectrophotometer against a blank made of methanol. Experiments were performed in triplicate, and the inhibition percentage (*I*) was calculated as follows: *I* (%) = ((OD_2_−OD_1_)_control_−(OD_2_−OD_1_)_sample_) x 100/(OD_2_−OD_1_)_control_

#### 2.5.2. Reducing Power Assay

The reducing power was performed according to a method described previously [20] using ascorbic acid as standard. Experiment was performed in triplicate, and optical densities were read at 700 nm.

#### 2.5.3. Lipid Peroxidation Assay

The effects of plant aqueous extract on lipid peroxidation were evaluated using the method described by Sawale et al. [21] except that the rat brain was used instead of the liver. Briefly, rat brain was freshly removed and homogenized in 0.15 M KCl solution using a tissue homogenizer (Potter-Elvehjem) to yield 10% brain homogenate. After centrifugation at 10000 rpm/min at 4°C (TGL-16 M, Loncare centrifuge) for 15 min, 500 *μ*l of supernatant was incubated in the presence or absence of the plant extract. Lipid peroxidation was initiated by adding 0.2 M ferric chloride. The reaction was stopped with an ice-cold HCl (0.25 N) solution containing 15% trichloroacetic acid and 0.38% thiobarbituric acid. Optical density was read at 532 nm. Gallic acid was used as positive control, and percentage inhibition was calculated as follows: inhibition of lipid peroxidation (%) = 100 × (1–OD sample)/OD control.

#### 2.5.4. AAPH-Induced Red Blood Cells Hemolysis

The antihemolytic effect of aqueous extract from *G*. *lucida* was assessed as previously reported [22] with some modifications. Blood samples were collected from healthy Wistar rats through the abdominal aorta into heparinized tubes. Red blood cells (RBCs) were separated from the plasma by centrifugation at 1500 rpm for 10 min and were subsequently washed three times with five volumes phosphate buffered saline (PBS, pH 7.4). Equal volume (100 *μ*L) of 5% RBC suspension, extract, and 200 mM AAPH (2′, 2′-azobis (2-methylpropionamidine) dihydrochloride) were mixed and incubated at 37°C for 3 h with gentle shaking. At the end of the incubation period, 4 mL of PBS was added to the reaction milieu, followed by centrifugation at 3500 rpm for 10 min. The optical density of the supernatant was read at 540 nm. To yield complete hemolysis, an aliquot of RBC suspension was treated with an equal volume of ice-cold distilled water. Control tubes were made of reaction milieu without extract or ascorbic acid, and the percentage inhibition was calculated as follows: *I* (%) = 100 × (1–OD sample)/OD control.

### 2.6. Evaluation of the Effect of *Garcinia lucida* on Adenine-Induced CKD

#### 2.6.1. Grouping and Dosing

Rats were randomly assigned into five groups of ten rats for each treatment as follows for four consecutive weeks:

- Group 1 served as control and received distilled water and saline (0.9 %)
- Group 2 received adenine (50 mg/kg) + distilled water
- Group 3 was treated with adenine (50 mg/kg) + captopril (20 mg/kg/day)
- Group 4 was treated with adenine (50 mg/kg) + aqueous extract (150 mg/kg/day)
- Group 5 was treated with adenine (50 mg/kg) + aqueous extract (300 mg/kg/days)

Chronic kidney disease was induced using a previously described method [23]. Adenine was daily injected (i.p) at the dose of 50 mg/kg for 4 consecutive weeks. Extract and captopril were administered orally by gavage during the same period.

The doses of extract used were calculated according to the traditional healer's instructions. The amount of extract solution recommended per day was taken from this traditional healer and evaporated at 40°C in an oven. Following this procedure, 1.452 g of powder was obtained and divided by 60 kg (reference human bodyweight). This corresponds to 24.20 mg/kg human dose. The equivalent dose for rats was calculated by multiplying the human dose (24.20 mg/kg) by 6.2 [24] resulting in a therapeutic dose of 150.04 mg/kg. The second dose of extract used in this study was obtained by multiplying the therapeutic dose by 2.

#### 2.6.2. Experimental Procedure

Blood pressure and heart rate were recorded at baseline and weekly using the noninvasive tail-cuff method (CODA, Kent Scientific Corporation). Bodyweight was also monitored weekly after the baseline value was taken. At the end of the treatment period, 24-hour urine samples were collected, centrifuged at 3500 rpm for 15 min at 4°C, aliquoted, and stored at −20°C for subsequent quantification of protein and creatinine. Following this, animals were anesthetized with sodium thiopental (50 mg/kg, i.p). Blood samples were collected through abdominal aorta in EDTA-coated tubes and centrifuged at 3500 rpm for 15 min at 40°C. The plasma obtained was kept at −20°C for urea, creatinine, ALT, and AST determination. The kidney and the heart were removed, freed of fats and connective tissues, washed in physiological saline, and weighed. The heart was dissected out, and pieces of each organ was fixed in 10% buffered formalin for routine hematoxylin and eosin staining technique, while the remaining part was stored at −20°C.

### 2.7. Biochemical Analysis

Urinary protein excretion was assessed using the Bradford method [25]. Urea, creatinine, ALT, and AST were assayed according to the manufacturer's instructions (SGM Italia). Creatinine clearance was used as an estimation of glomerular filtration rate (GFR) and was calculated as follows: GFR = *U* × V/*P* × 1440, where *U* is the urine creatinine, *V* is the 24 h urine volume, *P* is the plasma creatinine, and 1440 is time in seconds corresponding to 24 h.

The effect of the plant extract on oxidative stress parameters was determined by assaying enzymatic and nonenzymatic antioxidant status. A 15% tissue homogenate was prepared in 10 mM Tris-HCl buffer (pH 7.4) using a D‐160 handheld homogenizer (Loncare, China). Reduced glutathione (GSH) content was determined as described by [26]. Optical densities were read at 412 nm, and the amount of GSH was calculated using 1.36 × 10^4^ M^−1^·cm^−1^ as the molar extinction coefficient. Catalase (CAT) activity was evaluated according to the method of Sinha et al. [27]. A calibration curve for hydrogen peroxide (H_2_O_2_) was constructed, and optical densities were read at 570 nm. One unit of catalase activity was defined as the amount of enzyme required to decompose 1 micromole of H_2_O_2_ per minute. SOD activity was measured according to the method of [28].

### 2.8. Histopathological Assessment

The kidneys and the heart from all experimental groups were fixed in 10% buffered formalin, dehydrated in graded ethanol, cleared in xylene, and then embedded in paraffin. Sections of 5 *μ*m thickness were prepared and stained with hematoxylin and eosin. The stained sections were examined under a DN-107T light microscope for structural alterations.

### 2.9. Reagents

Adenine, aluminum chloride, potassium hydroxide, sodium nitrate, and Folin–Ciocalteu reagent were purchased from Carl-Roth (Germany). 2, 2-Diphenyl-1-picrylhydrazyl (DPPH), (2′, 2′-azobis (2-methylpropionamidine) dihydrochloride) (AAPH), gallic acid, quercetin, ascorbic acid, Tris, hydrogen peroxide, trichloroacetic acid, ferric chloride, and hydrogen chloride were purchased from Sigma–Aldrich (Germany). Captopril was purchased from Denk Pharma GmbH and Co., KG (Germany). Thiobarbituric acid was from Fluka. Sodium chloride and potassium chloride were purchased from BDH Chemicals Ltd. (England). Creatinine, urea, aspartate aminotransferase (AST), and alanine aminotransferase (ALT) kits were purchased at SGM Italia (Roma). Sodium thiopental was from Samarth Life Sciences Pvt Ltd. (Mumbai).

### 2.10. Statistical Analysis

Statistical analysis was performed using GraphPad Prism 5.03. All the results were expressed as mean ± standard error of the mean. IC_50_ was determined using the nonlinear regression curve following logarithmic transformation. For single time point data collection (organ weight and biochemical parameters), data were analyzed with one-way ANOVA followed by Tukey's post hoc test. For repeated measures (blood pressure and body gain weight), data were analyzed with two-way ANOVA with repeated measures followed by the Bonferroni post hoc test. Differences between means were considered significant at *p* < 0.05.

## 3. Results

### 3.1. Total Phenols and Flavonoids Content

The total amount of phenols and flavonoids in *G*. *lucida* extract is recorded in Table 1. It can be noticed that the total amount of phenolic compounds in the aqueous extract from the stem bark of *G*. *lucida* is high, while the total amount of flavonoids is low.

### 3.2. DPPH Radical Scavenging Activity

**Figure 1:**
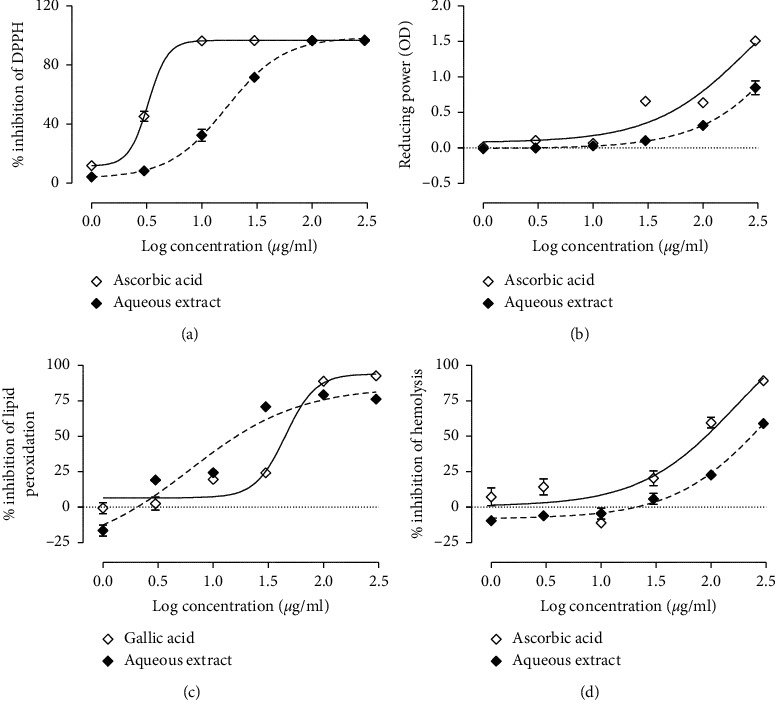
DPPH radical scavenging activity (a), total reducing power (b), antilipid peroxidation (c), and antihemolytic effects of the aqueous extract of *Garcinia lucida*.

As depicted in Figure 1(a), the plant extract exhibited a strong radical scavenging activity on DPPH, even though it was lower than that of ascorbic acid. The IC_50_ value of aqueous extract was 14.13 *μ*g/mL versus 3.27 *μ*g/mL for ascorbic acid (Table 1).

### 3.3. Reducing Power

Results concerning the reducing power showed that the aqueous extract from the stem bark of *G*. *lucida* exhibited a concentration-dependent reducing power, even though it was lower than the effect of ascorbic acid (Figure 1(b)).

### 3.4. Effects of Aqueous Extract of *Garcinia lucida* on Lipid Peroxidation

The effect of *G*. *lucida* extract on lipid peroxidation is depicted in Figure 1(c). It can be observed that the aqueous extract strongly inhibit lipid peroxidation more than gallic acid used here as a positive control. The effect of the plant extract was 7 times superior to that of gallic acid with respective IC_50_ value of 6.14 *μ*g/mL and 45.21 *μ*g/mL (Table 1).

### 3.5. AAPH-Induced Red Cells Hemolysis

The aqueous extract from *G*. *lucida* moderately protected red blood cells against hemolysis elicited by AAPH. The antihemolytic effect exhibited by the extract was observed only from the concentration of 30 *μ*g/mL. The IC_50_ value of aqueous extract was 349.70 *μ*g/mL versus 167.30 *μ*g/mL for ascorbic acid (Figure 1(d) and Table 1).

### 3.6. Effect of the Aqueous Extract of *Garcinia lucida* on Arterial Blood Pressure and Heart Rate

**Figure 2:**
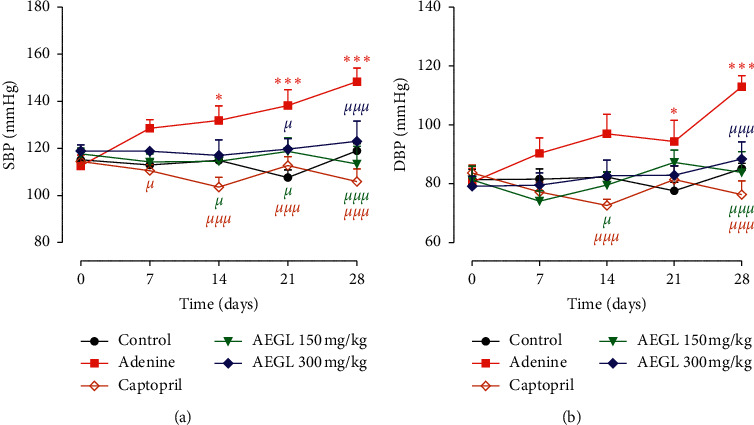
Effect of the aqueous extract of *Garcinia lucida* on systolic (a) and diastolic (b) blood pressure on adenine-induced chronic kidney disease. All values are expressed as mean ± SEM from 6 rats. ^*∗*^*p* < 0.05^,^^*∗∗*^*p* < 0.01, and ^*∗∗∗*^*p* < 0.001 compared to the control and ^*μ*^*p* < 0.05, ^*μμ*^*p* < 0.01, and ^*μμμ*^*p* < 0.001 compared to adenine. AEGL, aqueous extract of *Garcinia lucida.*

As shown in Figure 2, chronic administration of adenine induced a progressive and significant (*p* < 0.05 and *p* < 0.001) increase in systolic and diastolic blood pressure compared to the control group. Systolic blood pressure increased from 119.00 ± 3.05 mmHg in the control group to 148.30 ± 5.75 mmHg in the adenine group at the end of the treatment period. Diastolic pressure values changed from 85.08 ± 1.84 mmHg in control rats to 112.90 ± 3.84 mmHg in animals that were chronically administered with adenine. Concomitant administration of the plant extract at all tested doses or captopril significantly (*p* < 0.05 and *p* < 0.001) prevent the rise in blood pressure elicited by adenine. Blood pressure values in these groups were similar to that of the control group at the end of the experiment.

**Figure 3:**
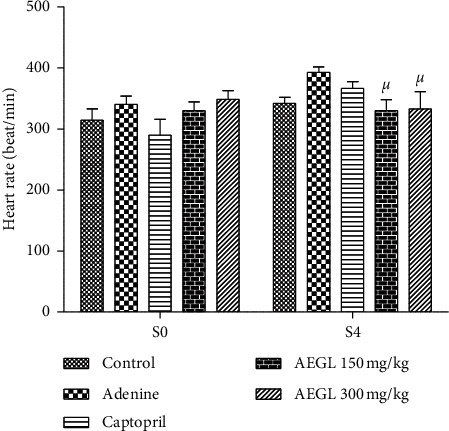
Effect of the aqueous extract of *Garcinia lucida* on heart rate in adenine-induced chronic kidney disease. All values are expressed as mean ± SEM from 6 rats. ^*μ*^*p* < 0.05 compared to adenine. AEGL, aqueous extract of *Garcinia lucida.*

No significant (*p* < 0.05) effect on heart rate was observed following adenine exposure. Nevertheless, heart rate was significantly low (*p* < 0.05) in rats that were concomitantly treated with adenine and plant extract compared to those that received adenine alone (Figure 3).

### 3.7. Effect of the Aqueous Extract of *Garcinia lucida* on Bodyweight and on Some Organs' Weight

As observed in Table 2, adenine administration resulted in bodyweight loss (*p* < 0.01) that was not inhibited by either captopril or the aqueous extract of *G*. *lucida*. Captopril and the extract at the dose of 150 mg/kg rather accentuated the weight loss.

Adenine treatment was also associated with a significantly increase in relative weight of the kidney (*p* < 0.001), heart (*p* < 0.01), and left ventricle (*p* < 0.001) compared to the control group. The most important organ mass increase was that of the kidney which enlarged more than double. The aqueous extract of *G*. *lucida* at the dose of 150 mg/kg completely abolished adenine-induced organ hypertrophy. Captopril acted similar to the plant extract at the dose of 150 mg/kg, except that it failed to prevent kidney hypertrophy (Table 2).

### 3.8. Effect of the Aqueous Extract of *Garcinia lucida* on Plasma Transaminases

**Figure 4:**
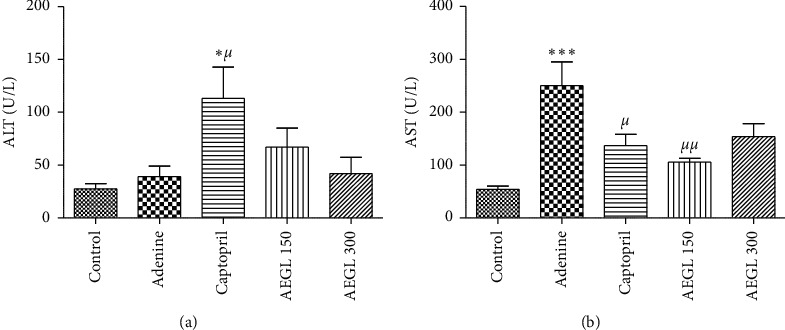
Effect of the aqueous extract of *Garcinia lucida* or captopril on rat plasma ALT (a) and AST (b) contents. All values are expressed as mean ± SEM from 6 rats. ^*∗*^*p* < 0.05,^*∗∗*^*p* < 0.01, and^*∗∗∗*^*p* < 0.001 compared to the control and ^*μ*^*p* < 0.05, ^*μμ*^*p* < 0.01, and ^*μμμ*^*p* < 0.001 compared to adenine. AEGL, aqueous extract of *Garcinia lucida.*

Long-term administration of adenine significantly (*p* < 0.001) increased AST levels but had no effect on the ALT level (*p* > 0.05) compared to the control. Plasma ALT and AST levels were kept near to the normal value following treatment with the plant extract. Captopril significantly (*p* > 0.05) rose ALT but lowered (*p* < 0.05) adenine-evoked AST increase (Figure 4).

### 3.9. Effect of the Aqueous Extract on Oxidative Stress Parameters

All oxidative stress markers assayed in this study (GSH, CAT, and SOD) were significantly (*p* < 0.05 and *p* < 0.001) reduced in the kidneys following chronic adenine administration. In the cardiac tissue, adenine also induced a decrease in these parameters, but only the reduction in SOD activity reached statistical significance (*p* < 0.05). The plant extract regardless of the dose used failed to inhibit the drop in renal GSH, eventhough this decrease was a little bit delay with the dose of 150 mg/kg (Table 3).

Concerning the activity of natural antioxidant enzymes, the extract was more effective in preventing the drop in CAT and SOD in the kidney. Moreover, the plant extract at the dose of 150 mg/kg boosted the activity of cardiac CAT, while that of cardiac SOD was blunted (Table 3).

### 3.10. Effects of the Aqueous Extract of *G*. *lucida* on Renal Function

**Figure 5:**
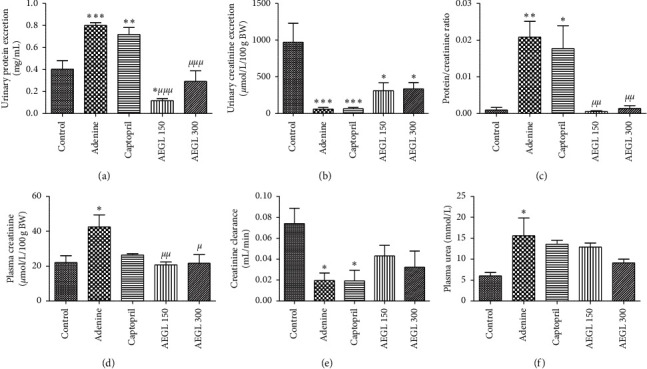
Effect of the aqueous extract of *Garcinia lucida* on renal markers in adenine-induced chronic kidney disease. All values are expressed as mean ± SEM from 6 rats. ^*∗*^*p* < 0.05, ^*∗∗*^*p* < 0.01, and^*∗∗∗*^*p* < 0.001 compared to the control and ^*μ*^*p* < 0.05, ^*μμ*^*p* < 0.01, and ^*μμμ*^*p* < 0.001 compared to adenine. AEGL, aqueous extract of *Garcinia lucida*.

As depicted in Figure 5, urinary protein excretion, protein/creatinine ratio, plasma creatinine, and serum urea were significantly increased (*p* < 0.05, *p* < 0.01, and *p* < 0.001) in adenine-treated rats, while urinary creatinine excretion and creatinine clearance declined significantly (*p* < 0.05) in the same animals. Creatinine clearance dropped from 0.07 ± 0.01 mL/min in control rats to 0.02 ± 0.01 mL/min in the adenine group (Figure 5(e)). All these parameters were greatly improved by the administration of the plant extract and particularly, urinary protein excretion and protein to creatinine ratio. Captopril to a lesser extent mitigates the effect of adenine on some markers of renal function, namely, urinary protein excretion, protein to creatinine ratio, and plasma creatinine.

### 3.11. Effects of the Aqueous Extract of *G*. *lucida* on Tissue Histology

**Figure 6:**
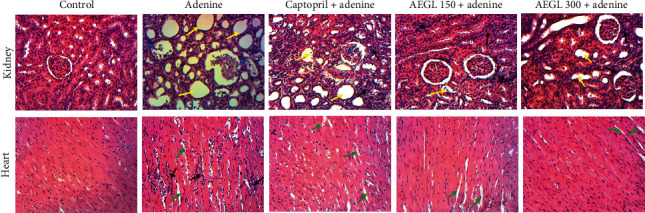
Representative photomicrographs showing the effect of the aqueous extract of *Garcinia lucida* (AEGL) on renal and cardiac histology in adenine-treated rats. The normal structure of the rat kidney is observed in the control group. Kidney from the adenine-treated group presented a general disorganization of the architecture, glomerular necrosis (blue arrow), and diffuse tubular dilatation (yellow arrow). The kidney from adenine + captopril-treated rat showed tiny glomerular degeneration and moderate tubular dilatation. In animals that received adenine + AEGL (150 mg/kg) or + AEGL (300 mg/kg), inflammatory cell infiltration (black arrow) and slight tubular dilatation were observed. Heart photomicrographs showed normal structure in the control group. Important inflammatory cell infiltration accompanied with increased intercellular spaces (green arrows) in adenine-treated animals. Scanty increase in intercellular space was observed in the adenine + captopril group. Extract-treated groups showed only increased intercellular spaces that was substantially reduced with AEGL at 300 mg/kg. Sections were stained with H&E. The kidney was observed at 400x, while the heart was pictured at 100x.

Figure 6 illustrates the effects of *G*. *lucida* aqueous extract on the histology of the kidney and heart stained with hematoxylin and eosin. The kidney of rats in the control group showed normal renal architecture without dilated tubules, glomerular necrosis, and leucocyte infiltration. Rats injected with adenine exhibited marked pathological alterations as severe and diffuse dilated tubules and glomerular necrosis. The oral administration of the plant extract improves the renal histology with the dose of 150 mg/kg being the most active. In fact, animals that received the lowest dose presented a renal structure with less dilated tubules and slight leucocyte infiltration. Animals treated concomitantly with adenine and captopril had a renal structure with marked tubular dilatation, leucocyte infiltration, and glomerular necrosis. Chronic administration of adenine was associated with cardiac tissue inflammation as evidenced by leucocyte infiltration and increased intercellular spaces. Scanty increase in intercellular space was observed in captopril-treated rats, while those which received the aqueous extract of *G*. *lucida* showed only slight increased intercellular spaces that were substantially reduced with the dose of 300 mg/kg.

## 4. Discussion

The present study was designed to evaluate the nephroprotective and cardioprotective effects of the aqueous extract from the stem bark of *G*. *lucida* in a rat model of adenine-induced chronic kidney disease (CKD). This experimental model was chosen in this study because it exhibits structural and functional alterations observed in CKD patients [29, 30].

Many reports have evidenced the role of oxidative stress in the pathogenesis and progression of adenine-induced CKD [23, 31]. Therefore, the antioxidant effect of *G*. *lucida* was evaluated in vitro and in vivo. Results from in vitro antioxidant tests showed that *G*. *lucida* extract exhibited a strong radical scavenging activity against DPPH. In alcoholic solution, DPPH forms a stable free radical, and the effect of antioxidant substances on this radical is thought to result from their hydrogen and/or their electron donating ability [32, 33].

It is well known that phenolic compounds and mostly flavonoids possess an ideal structure for free radical scavenging activity. In fact, the antioxidant properties of phenols arise from their high reactivity as hydrogen or electron donors [34, 35]. In this study, it has been noticed that the aqueous extract, which was rich in phenols, strongly scavenged DPPH radical and exhibited the highest reducing power. These findings suggest that the radical scavenging activity and the reducing power of *G*. *lucida* extract could be attributed to the presence of phenolic compounds.

One of the main mechanisms by which free radicals exert their deleterious effects on cells and organs is through lipid peroxidation of biological membranes. Since the extract used in this study showed interesting antiradical and reducing power, we tested their ability to inhibit lipid peroxidation initiated by ferric chloride through OH radical generation by Fenton's reaction. It was noticed that the aqueous extract from the stem bark of *G*. *lucida* strongly inhibited the peroxidation of brain's lipids. It is well known that radical scavenging activity and reduction of ferric ions are among the mechanisms by which antioxidants inhibit lipid [21, 36]. Thus, it can be suggested that aqueous extract from *G*. *lucida* inhibit lipid peroxidation by one of the above mechanisms.

Red blood cells are more prone to lipid peroxidation [37]. In an attempt to assess the antioxidant effect of *G*. *lucida* extracts on a living organism, they were tested on AAPH-induced red blood cells hemolysis. The results showed that aqueous extract exhibited a good antihemolytic effect, although these activities were observed at high extract concentrations (100 and 300 *μ*g/ml). AAPH induces red blood cells hemolysis by two mechanisms, namely, lipid peroxidation and protein oxidation [38]. Therefore, the antihemolytic effect of *G*. *lucida* extract matches well with the above demonstrated antilipid peroxidation activity.

The in vivo antioxidant effect of *G*. *lucida* was evaluated on a model of CKD using adenine. The results showed that adenine administration was associated with a reduction in GSH, CAT, and SOD supporting the findings of many other authors [23, 31, 39]. This reduction in the antioxidant defense mechanism was more important in the kidney than in the heart; suggesting that in this model, oxidative stress first developed in the renal tissue and secondary in the cardiac tissue. The concomitant administration of the plant extract mitigated the effect of adenine on the redox status of the renal tissue, especially at the dose of 150 mg/kg, suggesting that the plant possesses antioxidant effect. Besides, the dose of 150 mg/kg of the plant extract exerted its antioxidant activity in the heart by boosting the activity of CAT.

It was observed in this study that animals treated only with adenine progressively lost weight compared to healthy controls. This result is in accordance with previous works [23, 40, 41]. Loss in bodyweight in adenine-induced CKD was believed to be due to a decrease in food intake. Data on food intake in the adenine model are conflicting. Al Za'abi et al. [23] and Rahman et al. [41] observed no change in food intake, while Santos et al. [42] and Long et al. [43] noticed a significant decrease in food intake in adenine-treated rats. This discrepancy in food intake may be due to the route or the manner by which adenine is administered to animals. In the study of Al Za'abi et al. [23], adenine was injected intraperitoneally as in the present study. Rahman et al. [41] administered adenine by gavage, whereas Santos et al. [42] and Long et al. [43] incorporated adenine into feed. Mixing adenine to feed results in a reluctance to consume food because of adenine smell and taste [40], and in this case, bodyweight loss could be associated to low dietary intake. Eventhough food intake was not measured in this study, the abovementioned results suggest that other mechanisms than food intake may contribute to the bodyweight loss in this model. The administration of the plant extract did not prevent adenine-induced bodyweight loss. It instead aggravated this effect when used at the dose of 150 mg/kg, suggesting that *G*. *lucida* may possess a slimming effect.

It has been observed in this study that intraperitoneal injection of adenine alone increased proteinuria, protein to creatinine ratio, plasma creatinine, and serum urea but decreased creatinine excretion and creatinine clearance. These alterations are typical signs of CKD [1, 3] and clearly show that the induction of CKD was successful. Moreover, adenine induced kidney hypertrophy probably as a result of adenine crystal deposits. The treatment of rats with the plant extract totally or partially prevent all these abnormalities and suggest that the aqueous extract of *G*. *lucida* possesses metabolites with the nephroprotective effect.

Cardiovascular disease is one of the major causes of mortality and morbidity in patients with CKD, rather than from end-stage renal disease [39, 44]. Thus, in this study, the effect of *G*. *lucida* on the cardiovascular system was evaluated. The results showed that chronic adenine injection was associated with increased systolic and diastolic blood pressure, cardiac hypertrophy, cardiac inflammation, and increased cardiomyocyte intercellular space. Nguy et al. [29] and Kashioulis et al. [9] found that chronic adenine administration is associated with high blood pressure and aortic thickness. The thickening of the aortic vessel may contribute to increased vascular resistance. Increased vascular resistance might in turn increase blood pressure, and cardiac hypertrophy may develop as a consequence of increased cardiac workload. Treating animals with adenine plus the aqueous extract of *G*. *lucida* successfully prevented cardiac remodeling. This protective effect of the plant extract on cardiac architecture could be explained by its antihypertensive effect. Captopril also exhibited a cardioprotective effect suggesting that adenine-induced cardiovascular damages might be mediated by the renin-angiotensin system.

Histological analysis of sections from the renal tissue showed adenine-induced glomerular necrosis. This observation is supported by the high level of plasma AST. ALT levels did not change in the adenine group. However, Kim et al. [45] and Al Za'abi et al. [23] reported a significantly high levels of both AST and ALT following adenine exposition. The fact that ALT levels were not affected by adenine administration suggests that there was no liver damage in this model. The plant extract attenuated adenine-induced glomerular necrosis and increased plasma AST suggesting an antinecrotic potential. Angiotensin-converting enzyme inhibitors (ACE I) including captopril are recommended for the management of CKD in nondiabetic adults [2]. Therefore, it was expected that captopril improves renal alterations elicited by adenine. Surprisingly, the results obtained in this study clearly showed that captopril failed to improve adenine-induced CKD. Instead, it tends to worsen the effect of adenine. The nephrotoxicity associated with ACE I has been extensively reported by some authors [46–48]. Hence, the results obtained with captopril may be due to that nephrotoxic effect.

## 5. Conclusion

In the light of what precedes, it can be concluded that the aqueous extract from the stem bark of *G*. *lucida* exhibits in vitro and in vivo antioxidant activities. This antioxidant activity could be ascribed to its content in phenolic compounds. Besides, the cardioprotective and nephroprotective effects of *G*. *lucida* may be ascribed to its antihypertensive and antioxidant activities.

## Figures and Tables

**Table 1:** Total phenols and flavonoids contents and IC_50_ values of the in vitro antioxidant effects of the aqueous extract from the stem bark of *Garcinia lucida*.

	Total phenols (mg GAE/g extract)	Flavonoids (mg QE/g extract)	IC_50_ values (*μ*g/mL)		
			DPPH	AAPH	Lipid peroxidation

Aqueous extract	258.10 ± 14.82	6.09 ± 0.07	14.13	349.70	6.14
Ascorbic acid	—	—	3.27	167.30	—
Gallic acid	—	—	—	—	45.21

Experiments were performed in triplicate. GAE, gallic acid equivalent; QE, quercetin equivalent.

**Table 2:** Effect of aqueous extract of *Garcinia lucida* (AEGL) treatments on bodyweight gain and on relative kidney, heart, and left ventricle weight in rats with adenine-induced chronic kidney disease.

Experimental groups	Body weight gain (%)	Kidney weight (g/cm tibia length)	Heart weight (g/cm tibia length)	Left ventricle weight (g/cm tibia length)
Control	11.03 ± 0.80	0 .37 ± 0.01	0.18 ± 0.00	0.14 ± 0.00
Adenine	−3.37 ± 4.07^∗∗^	0.87 ± 0,09^*∗∗∗*^	0.22 ± 0.01^*∗∗*^	0.17 ± 0.01^*∗∗∗*^
Adenine + captopril (20 mg/kg)	−8.80 ± 2.43^*∗∗∗*^	0.65 ± 0,06^∗∗∗^	0.18 ± 0.00^*μμ*^	0.13 ± 000^*μμμ*^
Adenine + AEGL (150 mg/kg)	−13.34 ± 3.39^*∗∗∗*^	0.45 ± 0.01^*μμμ*^	0.19 ± 0.01	0.14 ± 0.00^*μμ*^
Adenine + AEGL (300 mg/kg)	−2.56 ± 1.19^*∗*^	0.67 ± 0.07^*∗∗∗*^	0.20 ± 0.01	0.15 ± 0.00

All values are expressed as mean ± SEM from 6 rats. ^*∗*^*p* < 0.05,^*∗∗*^*p* < 0.01, and ^*∗∗∗*^*p* < 0.001 compared to control and ^*μ*^*p* < 0.05, ^*μμ*^*p* < 0.01, and^*μμμ*^*p* < 0.001 compared to adenine. Data were analyzed with one-way ANOVA, followed by Tukey's multiple comparison test.

**Table 3:** Effect of the aqueous extract of *Garcinia lucida* (AEGL) or captopril on renal and cardiac-reduced glutathione (GSH), catalase (CAT), and superoxide dismutase (SOD).

Parameters	Organs	Control	Adenine (50 mg/kg/days, i.p)			
			Distilled water	Captopril	AEGL, 150 mg/kg	AEGL, 300 mg/kg

GSH (*μ*mol/mg of tissue)	Kidney	2.40 ± 0.30	0.44 ± 0.14^*∗∗∗*^	0.74 ± 0.23^*∗∗*^	0.96 ± 0.26^*∗∗*^	0.49 ± 0.16^*∗∗∗*^
	Heart	1.38 ± 0.11	1.05 ± 0.16	0.79 ± 0.22^*∗*^	0.67 ± 0.24^∗^	1.18 ± 0.26

CAT (UI/mg of protein)	Kidney	223.20 ± 13.22	66.65 ± 19.59^*∗∗∗*^	124.00 ± 13.99^*∗∗∗*^^*μμ*^	170.40 ± 8.57^*∗∗*^^*μμ*^	133.50 ± 16.10^*∗∗*^^*μμ*^
	Heart	160.30 ± 21.61	108.00 ± 11.60	243.50 ± 31.01^*μμ*^	328.10 ± 74.96^*∗*^^*μ*^	114.10 ± 12.93

SOD (U/g of protein)	Kidney	8.37 ± 1.94	2.39 ± 0.45^*∗*^	5.34 ± 1.56	5.06 ± 1.25^*μμ*^	6.03 ± 1.58^*μ*^
	Heart	7.76 ± 1.18	3.95 ± 0.70^*∗*^	2.36 ± 0.26^*∗∗*^	2.32 ± 0.54^*∗∗*^	2.79 ± 0.91^*∗*^

All values are expressed as mean ± SEM from 6 rats. ^*∗*^*p* < 0.05, ^*∗∗*^*p* < 0.01, and ^*∗∗∗*^*p* < 0.001 compared to control and ^*μ*^*p* < 0.05, ^*μμ*^*p* < 0.01, and ^*μμμ*^*p* < 0.001 compared to adenine. Data were analyzed with one-way ANOVA, followed by Tukey's multiple comparison test.

## Data Availability

The data used to support the findings of this study are available from the corresponding author upon request
